# A network pharmacological-based study of the mechanism of Liuwei Dihuang pill in the treatment of chronic kidney disease

**DOI:** 10.1097/MD.0000000000033727

**Published:** 2023-05-12

**Authors:** Xi Xie, Hongjun Lou, Ye Shi, Guang Gan, Hanqing Deng, Xinwei Ma, Mingfang Meng, Xi Gao

**Affiliations:** a The First Clinical Medical College of Heilongjiang University of Traditional Chinese Medicine, Harbin, China; b The First Affiliated Hospital of Heilongjiang University of Traditional Chinese Medicine, Harbin, China; c College of Integrated Chinese and Western Medicine, Hunan University of Traditional Chinese Medicine, Changsha, China; d The First Clinical Medical College of Hunan University of Traditional Chinese Medicine, Changsha, China.

**Keywords:** chronic kidney disease, Liuwei Dihuanng pill, mechanism of action, molecular docking, network pharmacology

## Abstract

Chronic kidney disease (CKD) is a progressive disease that poses a huge economic burden to society. Liuwei Dihuanng pill is an effective treatment for chronic kidney disease, but its treatment mechanism is unclear. The rapid development of network pharmacology has provided new strategies for studying Chinese medicine.

**Method:**

The traditional Chinese medicine systems pharmacology database and analysis platform was used to obtain the bioactive components and targets of Liuwei Dihuanng pill. The sources for the CKD-related targets were then obtained from the Genecards, OMIM, TTD, and DisGeNET databases. R was used to identify the intersecting genes for Liuwei Dihuang pill and CKD-related targets. Analysis of protein-protein interactions (PPI) was performed using STRING, and PPI networks and drug-component-target networks were constructed using Cytoscape software. Kyoto encyclopedia of genes and genomes pathway and gene ontology enrichment analyses were performed using R. Finally, molecular docking was performed to determine the binding activity between bioactive components and the targets.

**Result:**

After screening and data de-duplication of 74 active components, 209 drug targets, and 14,794 disease targets, a total of 204 drug-disease targets were acquired. Subsequently, a drug-component-target network and PPI network were established. The primary components of Liuwei Dihuang pill included quercetin, stigmasterol, kaempferol, beta-sitosterol, tetrahydroalstonine, kadsurenone, hederagenin, hancinone C, diosgenin, and sitosterol. In addition, JUN, AKT1, TP53, RELA, MAPK1, FOS, TNF, IL6, ESR1, and RXRA were identified as the main targets. Gene ontology function enrichment analysis revealed that these targets were involved in reactive oxygen species metabolic processes, responses to metal ions and to chemical stimuli, G protein-coupled amine receptor activity, and nuclear factor receptor activity. Kyoto encyclopedia of genes and genomes enrichment analysis showed that these targets were involved in the AGE-RAGE signaling pathway, IL-17 signaling pathway, TNF signaling pathway, and so on. Molecular docking results indicated good binding activity between the core targets and core components.

**Conclusion:**

The potential mechanism of Liuwei Dihuanng pill in the treatment of CKD was preliminarily discussed in this study, providing a theoretical basis and evidence for further experimental research.

## 1. Introduction

Chronic kidney disease (CKD) is characterized by impaired kidney function or structural damage persisting for more than 3 months, resulting in a decline in overall health. The condition is typically diagnosed based on a glomerular filtration rate below 60 mL/min-1.73 m^2^ or a urine albumin to creatinine ratio of 30 mg/g or higher.^[1]^ CKD represents a growing global public health issue, imposing a significant economic burden on society, as current statistics estimate a prevalence rate of approximately 11% to 13% for this condition.^[2,3]^ The progressive decline in the glomerular filtration rate (GFR) causes structural, functional, and molecular alterations in the renal unit, which is a distinctive feature of CKD. The decline in GFR triggers compensatory hypertrophy of the glomerulus, which can eventually lead to glomerulosclerosis and tubular necrosis. This process ultimately results in renal failure when the GFR falls below 15 mL/min·1.73 m^2^ or when the urine protein exceeds 300 mg/g.^[4,5]^ Hypertension, diabetes, atherosclerosis, and autoimmune diseases are traditional risk factors for the development of CKD. Poor lifestyle habits, such as smoking, alcohol consumption, and a high purine diet, are also considered to contribute to the development of these conditions.^[6]^ nontraditional risk factors include pregnancy, kidney stones, nephrotoxic drugs, infectious diseases, and acute kidney injury (AKI).^[7]^ The prevention of CKD is typically approached at 3 distinct levels. The first level involves establishing a healthy lifestyle, while the second level focuses on monitoring risk factors that may increase the likelihood of developing CKD. The third level centers around controlling blood pressure and proteinuria as a means of preventing or delaying the progression of CKD.^[8]^ The available treatments for CKD are currently quite limited, primarily focusing on strategies such as controlling protein intake, reducing blood sugar and blood lipids, inhibiting the renin-angiotensin-aldosterone system, and addressing poor lifestyle habits to slow the progression of the disease. Among these modalities, inhibition of the renin-angiotensin-aldosterone system is generally considered the most important intervention.^[7]^ The pathogenesis of CKD is complex, and existing studies suggest the involvement of the Wntβ-catenin signaling pathway, Transforming Growth Factor-β (TGFβ)-samds signaling pathway, Hippo/YAP signaling pathway, tumor necrosis factor (TNFα), HF-1, and P53.^[9–11]^

Liuwei Dihuanng pill is a prescription created by Yi Qian, a famous Chinese medical practitioner from the Northern Song Dynasty. Clinical studies have shown that Liuwei Dihuang pill (LWDH) can effectively increase GFR (*P *< .05) and reduce the ratio of urinary albumin to creatinine (*P *< .05) in patients with CKD.^[12]^ Furthermore, another study found that patients with CKD who used LWDH as adjuvant therapy exhibited a lower incidence of ischemic stroke (*P *< .05). Liu et al^[13]^ used LWDH to treat osteoporotic rats with diabetic nephropathy, demonstrating that LWDH significantly reduced the pathological damage of renal tissues, such as glomerular hypertrophy, tubular necrosis, and basement membrane thickening, and decreased serum TNF, IL-6, IL-8, and IL-1 levels in the model group (*P* < .05). All the above studies showed that LWDH is effective in treating CKD. However, the components of LWDH are complex and have many action targets. Therefore, further research on the bioactive components of LWDH in CKD treatment should be carried out to investigate the mechanism of action of LWDH in CKD. The concept of network pharmacology was first introduced by Andrew L Hopkins.^[14]^ Disease development is not mediated by a single target or signaling pathway, but involves multiple targets; similarly, a drug achieves its therapeutic effect through multiple active ingredients acting on multiple targets. Chinese medicines often involve multiple active components, signaling pathways, and active targets for treating diseases. The rapid development of network pharmacology provides new strategies for studying Chinese medicines, which can help elucidate the pharmacological mechanisms of the active components of Chinese medicines at the molecular biology level.

In this study, a network pharmacological model of LWDH was built to methodically investigate their mechanism in CKD treatment. Our study may reveal the mechanisms underlying CKD development. Additionally, molecular docking was performed to validate the model by matching the targets of CKD with the active components of LWDH.

## 2. Materials and methods

### 2.1. Ingredient collection and target prediction of LWDH

As an open-source database, Traditional Chinese Medicine Systematic Pharmacology Database and Analysis Platform (TCMSP) allows for the analysis of active ingredients, pharmacokinetics, pharmacometabolomic, and genomics of Chinese medicines.^[15]^ In the TCMSP database, oral bioavailability (OB) ≥ 30% (indicates the rate and degree of drug entering the human blood circulation) and drug-likeness (DL) ≥ 0.18 (indicates the similarity of a compound to a known drug) were set as screening conditions to identify the bioactive compounds of each herbal medicine in LWDH, with their corresponding targets of action.

### 2.2. Predicting the targets of CKD

“Chronic kidney disease” was used as the search term and CKD-related protein targets were collected from the databases shown in Table 1. The results of the 4 disease database searches were merged and duplicate values were removed to obtain CKD-related targets.

**Table 1:** Database information of CKD target sources.

Database name	Internet site
GeneCards	(https://www.genecards.org)
OMIM	(https://www.omim.org/)
Therapeutic Target Database	(http://db.idrblab.net/ttd/)
DisGeNet	(https://www.disgenet.org/)

CKD = chronic kidney disease.

### 2.3. Protein–protein interaction (PPI) network construction

Venn diagrams were plotted using R (https://www.r-project.org) to obtain the intersection of LWDH and CKD targets. Subsequently, the intersecting protein targets were uploaded to the STRING database (https://string-db.org) to construct a PPI network. The species was set to “human,” the confidence level was set to greater than 0.9 (the highest confidence level), and the other setting options were kept at their default values. Next, the PPI results were exported in TSV format and a visualization network was constructed in Cytoscape. According to the parameters of the network, the critical targets of LWDH in the treatment of CKD were predicted.

### 2.4. Construction and analysis of drug-component-target network

The active ingredients and targets were imported into Cytoscape to construct a drug-ingredient-target network to clarify the relationship between the active ingredients of LWDH and the drug-disease target. In addition, the core targets of LWDH were determined by analyzing various network parameters.

### 2.5. KEGG pathway and GO function enrichment analyses

To elucidate the target protein biological functions and signaling pathways between LWDH and CKD, the potential therapeutic targets were imported into R Studio software and the clusterProfiler package was used to perform gene ontology (GO) enrichment analysis and Kyoto Encyclopedia of Genes and Genomes (KEGG) signaling pathway enrichment analysis on the potential targets. *P* < .05 was set for Homo sapiens, and the results were visualized using the ggplot2 package.

### 2.6. Molecular docking

The core targets obtained in section 2.3 and the bioactive components obtained in 2.4 were selected for molecular docking to evaluate the binding activity. The 3D structure of the target proteins was acquired from the PDB database (https://www.rcsb.org), and the 3D structure of the drug’s active ingredient was obtained from the TCMSP. PyMOL was used to remove water molecules the target protein and basic ligands before importing it into AutoDock Tools to perform hydrogenation, charge calculation, and non-polar hydrogen combination. Molecular docking was performed using AutoDock Vina, and the results were visualized using PyMOL.

## 3. Results

### 3.1. Ingredient collection and target prediction of LWDH

In the TCMSP database, a total of 508 bioactive ingredients were identified from the 6 herbs of LWDH, and 74 active components were obtained with *OB* ≥ 30 and *DL *≥ 0.18 as the screening conditions. These components included 15 components of Poria, 11 components of Cortex Moutan, 16 components of Rhizoma Dioscoreae, 2 components of Radix Rehmanniae Praeparata, 20 components of Fructus Corni, and 10 components of Rhizoma Alismatis. Based on the obtained active ingredients, 209 potential targets of LWDH were retrieved.

### 3.2. Predicting the targets of CKD

The number of targets for CKD obtained from the Genecard, OMIM, TTD, and DisGeNet databases were 14,364, 489, 35, and 1074, respectively. Finally, 14,794 targets were obtained after removing duplicate values.

### 3.3. PPI network construction

**Figure 1.**
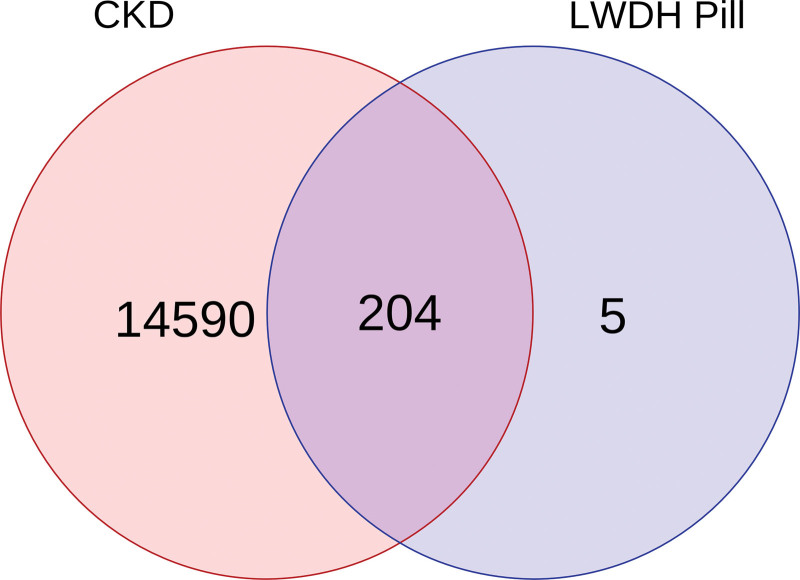
Venn diagram of CKD-related targets and LWDH targets. CKD = chronic kidney disease, LWDH = Liuwei Dihuang pill.

**Figure 2.**
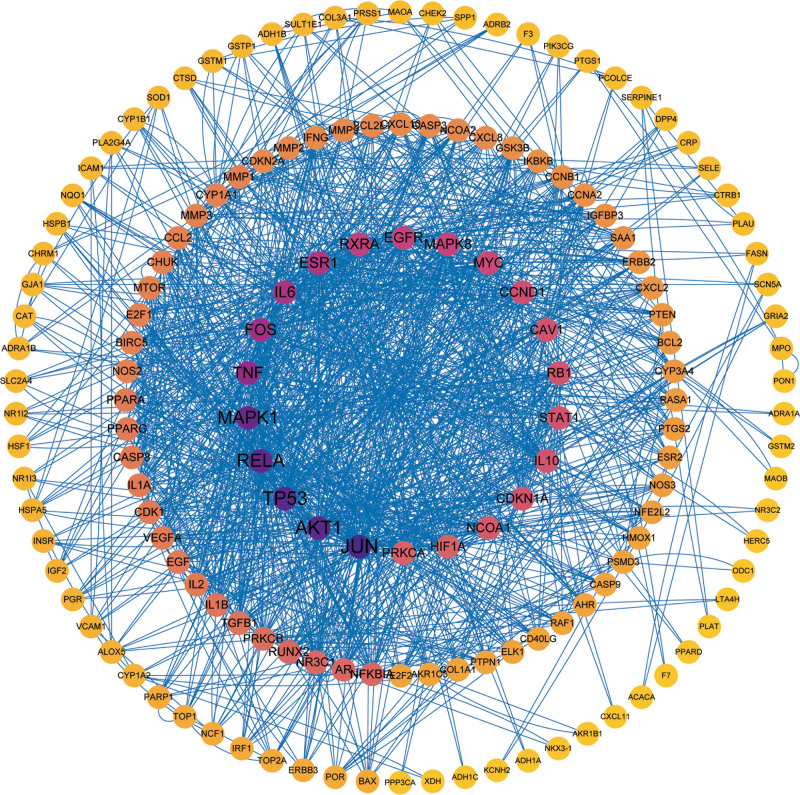
PPI network of LWDH and CKD proteins. The outermost circle indicates degrees 0–10, the middle circle indicates degrees 11–30, and the innermost circle indicates degrees 31–100. CKD = chronic kidney disease, LWDH = Liuwei Dihuang pill.

**Figure 3.**
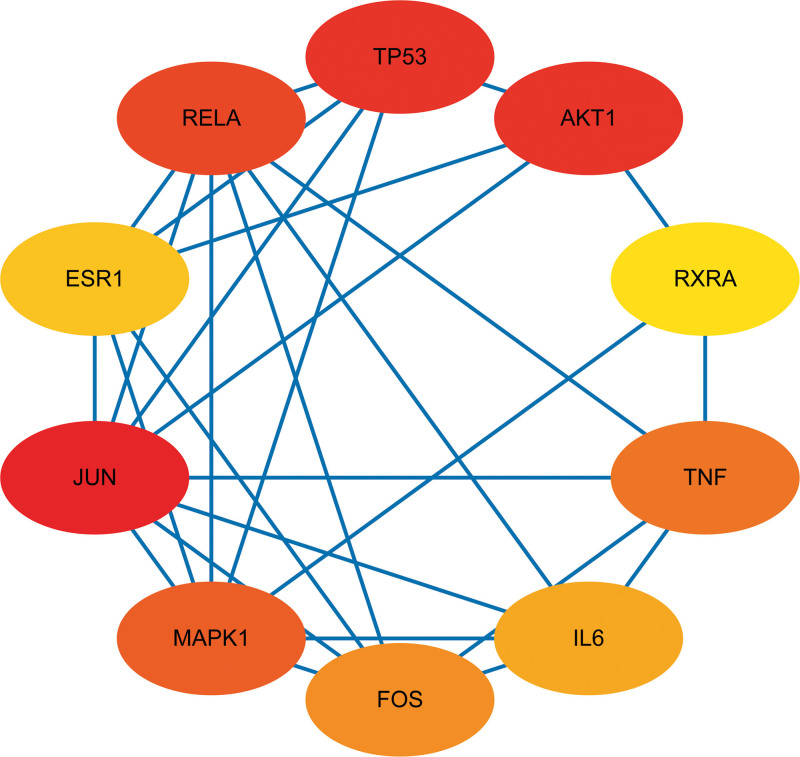
Hub gene target network of LWDH-CKD. The top 10 central gene networks of the target proteins were analyzed by the Degree algorithm using the Cytoscape plugin Cytohubba, where the gray values indicate the importance of the genes in the network. CKD = chronic kidney disease, LWDH = Liuwei Dihuang pill.

Using R, the LWDH targets were mapped to the CKD targets, yielding a total of 204 LWDH-CKD targets (Fig. 1). The intersecting protein targets were then uploaded to the STRING database to construct a PPI network. Visual analysis by Cytoscape revealed that the network had 156 nodes and 1292 edges (Fig. 2). The network was analyzed with Cystoscape’s network analysis tool and the top 10 targets were selected as the core targets of LWDH to treat CKD based on the degree value, including JUN, AKT1, TP53, RELA, MAPK1, FOS, TNF, IL6, ESR1, and RXRA (Fig. 3).

### 3.4. Construction and analysis of drug-component-target network

**Figure 4.**
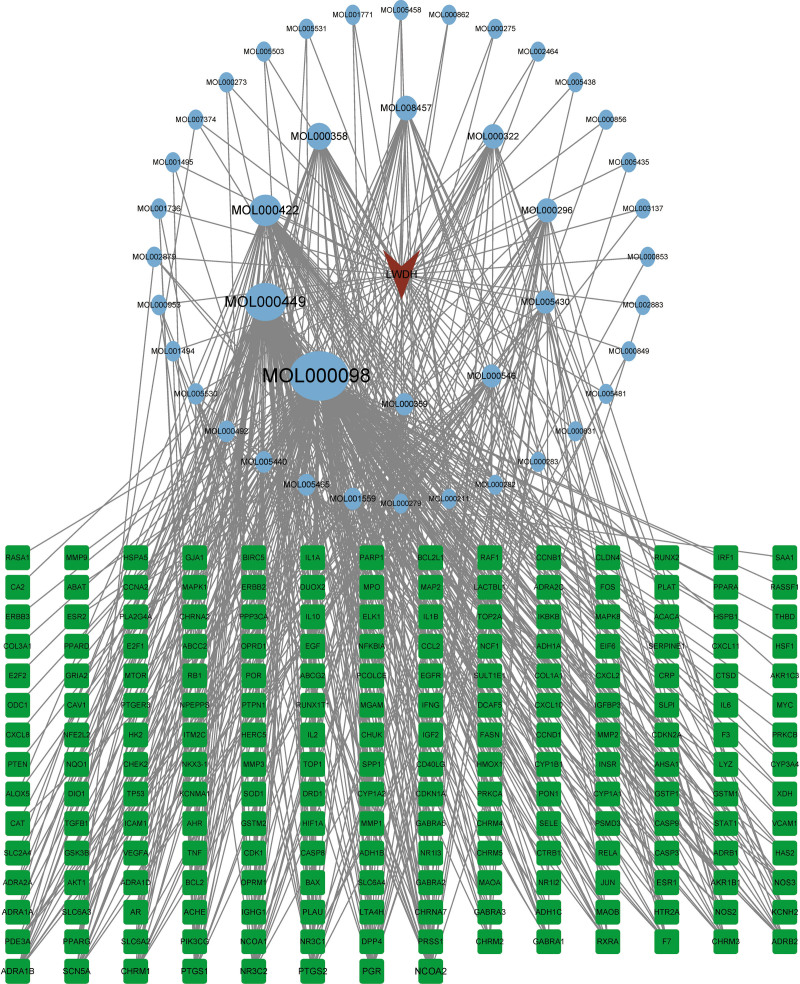
Drug-component-target network. (Blue circular nodes indicate components, green square nodes indicate drug-disease targets, and V-shaped nodes indicate LWDH; the size of the nodes reflects their degree). LWDH = Liuwei Dihuang pill.

A drug-component-target network with 247 nodes and 572 edges was obtained after screening the active components corresponding to the 204 drug-disease targets, yielding a total of 42 components (Fig. 4). This network was created by entering the connections between these active components and drug-disease targets into the Cystoscape. Subsequently, the betweenness centrality, closeness centrality, and degree of each component was calculated, and the network feature parameters were analyzed using Cystoscape’s Network Analyzer analysis program. The bioactive components were ranked by degree, with the top 10 being quercetin, stigmasterol, kaempferol, beta-sitosterol, tetrahydroalstonine, kadsurenone, hederagenin, hancinone C, diosgenin, and sitosterol. Table 2 displays their 2D structures, OB, DL, degree, betweenness centrality, and closeness centrality.

**Table 2:**
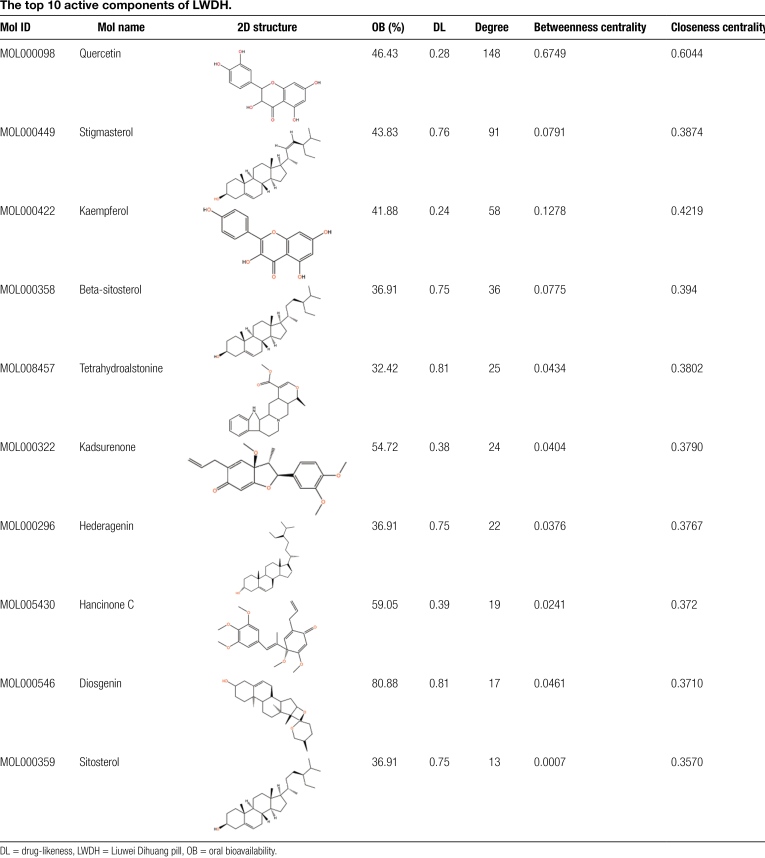
The top 10 active components of LWDH.

### 3.5. KEGG pathway and GO function enrichment analyses

**Figure 5.**
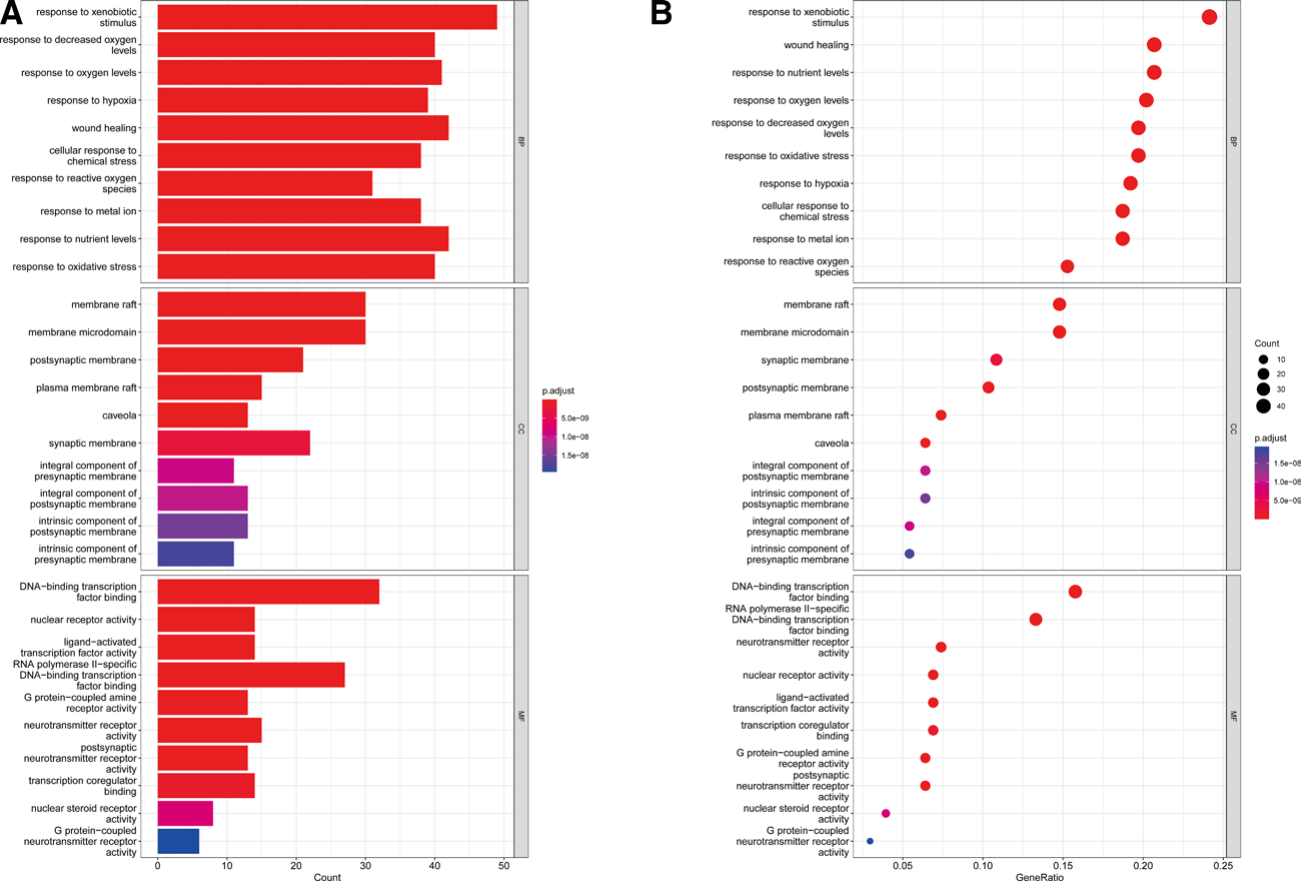
Results of GO enrichment analysis. A: Barplot; B: Dotplot. The X-axis represents the significant increase in counts or GeneRatio for these terms. Y-axis represents the “biological process,” “cellular component,” and “molecular function” categories in the GO enrichment analysis of target genes. “Categories in the GO enrichment analysis”. GO = gene ontology.

**Figure 6.**
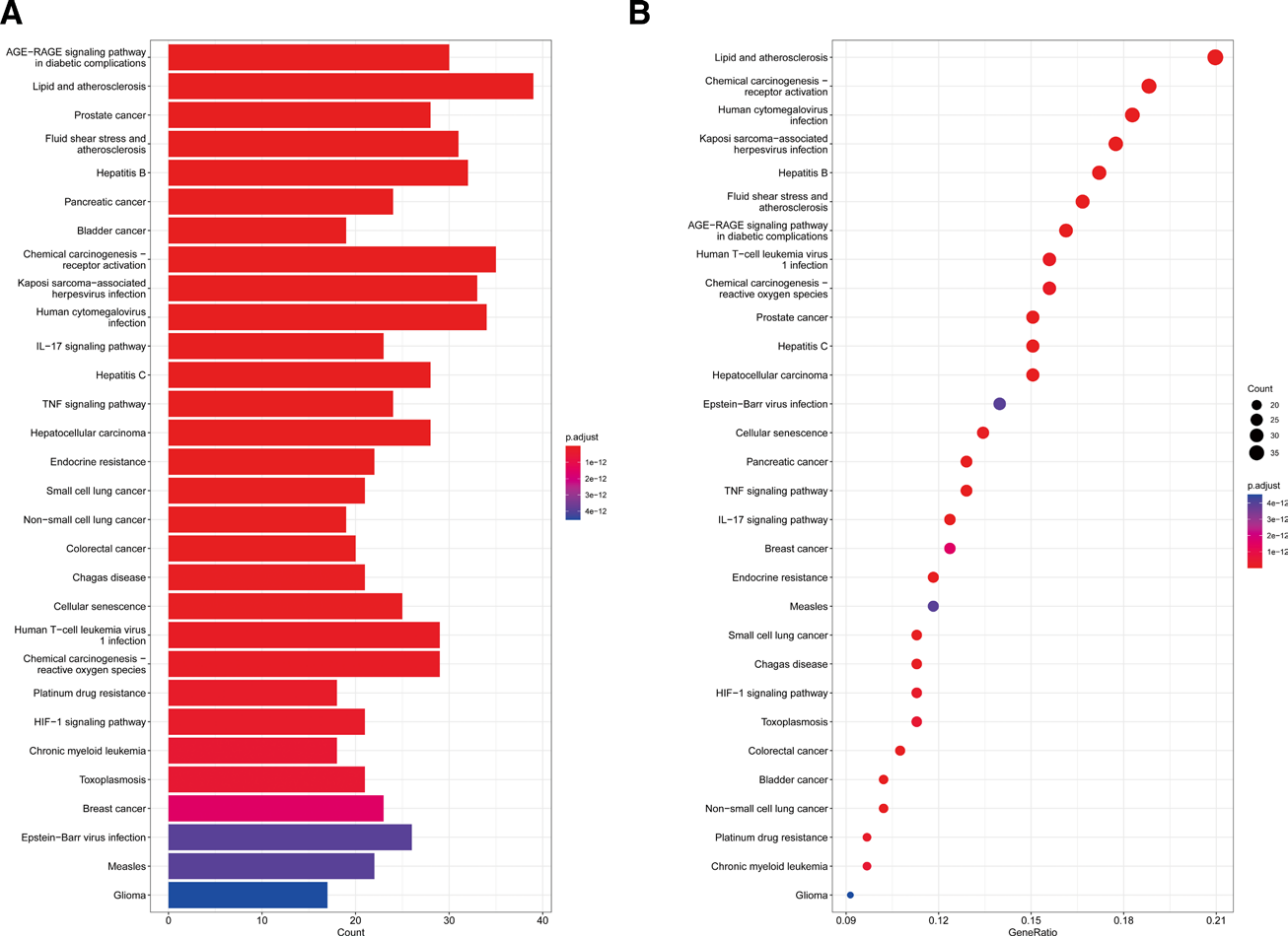
Results of KEGG enrichment analysis. A: Barplot; B: Dotplot. The X-axis represents the significant increase in counts or GeneRatio for these terms. Y-axis represents the main pathway. KEGG = Kyoto Encyclopedia of Genes and Genomes.

R was used for GO enrichment analysis of the above drug-disease targets, including GO biological processes, GO cellular components, and GO molecular functions (MF). Then, bubble plots were prepared for additional analysis using the first 10 results for each item that was preserved (Fig. 5). These targets were prevalent in many biological processes, revealing that LWDH can control a variety of biological functions in CKD treatment. Reactive oxygen metabolic activities, reactions to metal ions, and reactions to chemical stimuli were the biological processes most closely linked to CKD. Molecular functions that were closely related to CKD included G protein − coupled amine receptor activity, nuclear steroid receptor activity, DNA − binding transcription factor binding, nuclear receptor activity, and so on. The first 30 results of the KEGG enrichment analysis project were recorded (Fig. 6), mainly including the Advanced-glycation end products (AGES) − receptor Advanced Glycation Receptor Products (RAGEs) signaling pathway in diabetic complications, multiple virus infections, IL − 17 signaling pathway, TNF signaling pathway, etc.

### 3.6. Molecular docking

**Figure 7.**
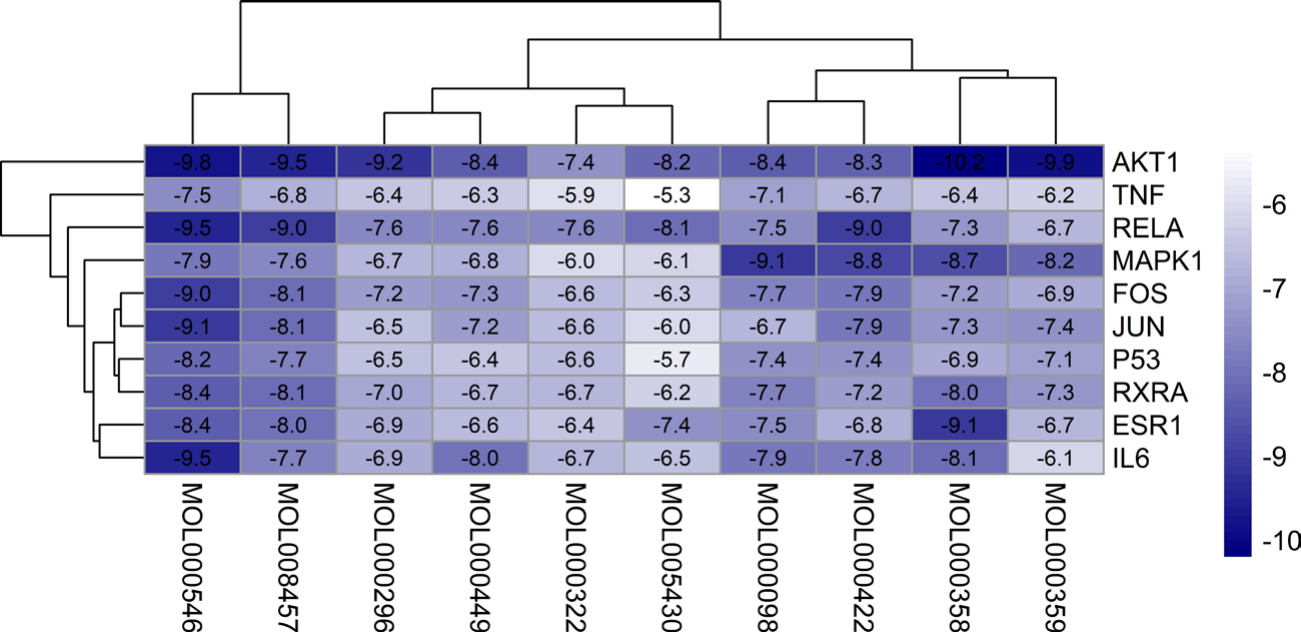
The heatmap of molecular docking binding energy. The Y-axis indicates the name of the core target, the X-axis indicates the MOL ID of the core active ingredient of LWDH, and the data in the heat map represent the binding energy of molecular docking. The lower the binding energy, the darker the cell color. LWDH = Liuwei Dihuang pill.

**Figure 8.**
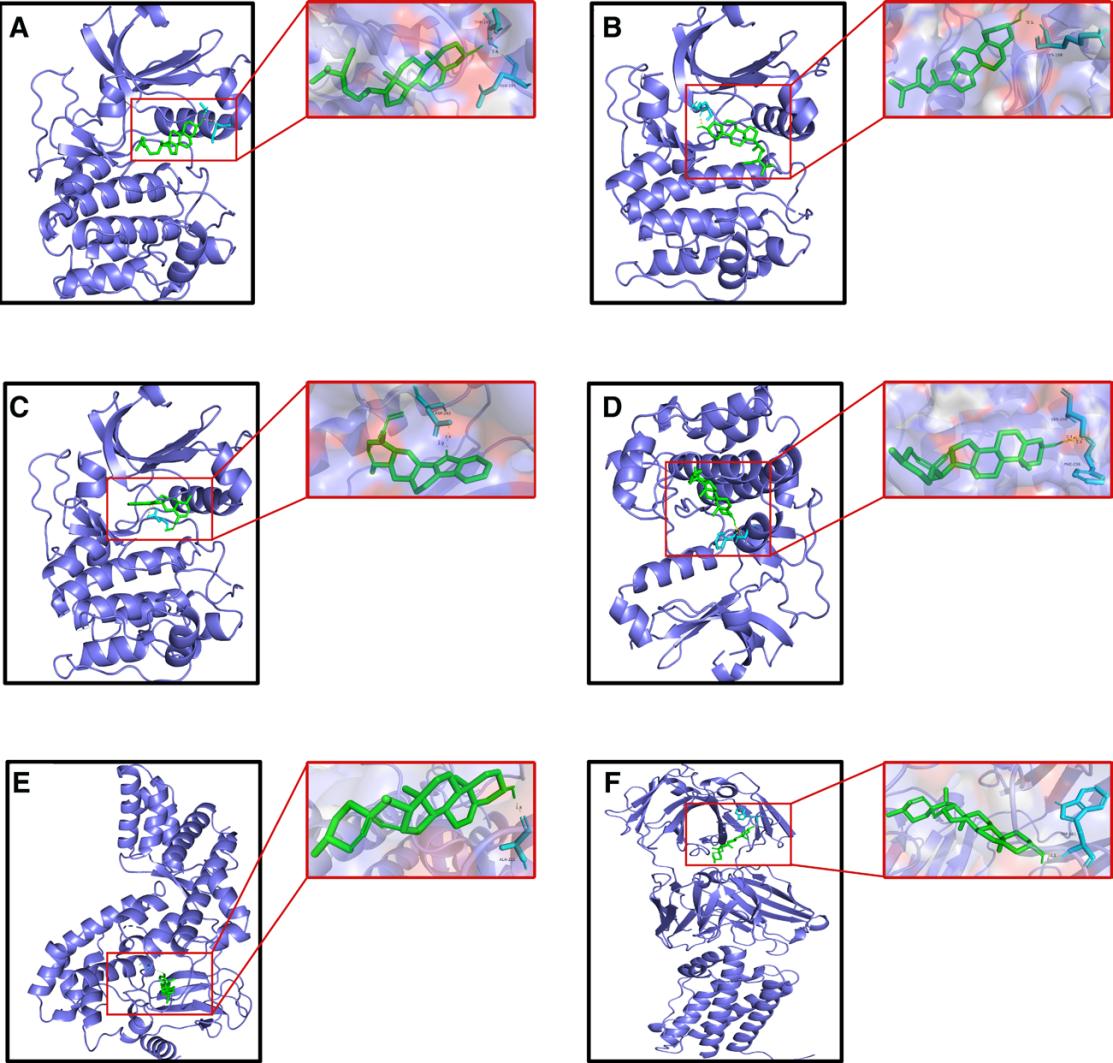
Visualization of molecular docking results. (A) AKT1-Beta-sitosterol; (B) AKT1-sitosterol; (C) AKT1-Tetrahydroalstonine; (D) AKT1-Diosgenin; (E) RELA-Diosgenin; (F) IL6-Diosgenin. Green sticks represent central proteins, cyan sticks represent ligands, and yellow sticks represent hydrogen bonds. The length of the hydrogen bond is indicated by a number next to the stick.

Molecular docking was performed and the core components of LWDH were visualized with the core drug-disease targets. The top 10 active components were screened as ligands and the top 10 central LWDH-CKD targets as binding sites for molecular docking. The binding energy is an important indicator for evaluating whether the active ingredient and the target have good binding activity. The lower the binding energy, the stronger the binding activity. Using R, a heat map of the molecular docking binding energy was plotted (Fig. 7). The results revealed that all binding energies were <5 kJ/mol, indicating good binding activity of the bioactive components and the core targets. Finally, Pymol was used to visualize and analyze the docking results for binding energies ≤9.5 kJ/mol (Fig. 8).

## 4. Discussion

Network pharmacology is a novel drug design approach that aims to identify disease-target-drug associations systematically and comprehensively. This method provides a holistic understanding of complex drug synergies in the human body by observing the effects and mechanisms of drug-disease interactions at the network level. Network pharmacology has allowed the discovery of active ingredients and mechanisms of action in Chinese medicine in treating various ailments, which has transformed the study of Chinese medicine from an empirical to evidence-based practice.^[16]^

In this study, open-source databases were utilized to gather information on CKD and identify the active components of LWDH for CKD treatment. The interactions between LWDH and CKD protein targets were predicted and molecular docking analysis was performed. The PPI results suggest that LWDH can act on CKD through multiple targets and pathways, with the top 10 targets being JUN, AKT1, TP53, RELA, MAPK1, FOS, TNF, IL6, ESR1, and RXRA. KEGG enrichment analysis revealed that LWDH exerts its therapeutic effects on CKD by regulating the AGE-RAGE signaling pathway, IL-17 signaling pathway, TNF signaling pathway, HIF-1 signaling pathway, and multiple viral infections. Based on the analysis of central targets and KEGG enrichment results, we can attribute the mechanism of LWDH intervention in CKD to the following factors.

Renal fibrosis is now acknowledged as a critical factor contributing to the irreversible progression of CKD, and multiple core targets are involved in this process. Among the members of the AKT superfamily, AKT1 has been extensively researched.^[17]^ Renal fibrosis is the primary pathological process leading to the progression of AKI to CKD. Recent research indicates that mice with AKT1 gene knockdown exhibit lower levels of renal fibrosis after unilateral ischemia-reperfusion injury (IRI) compared to wild-type mice. This finding highlights the crucial role of AKT1 in the development of renal fibrosis and suggests that AKT1 may be a significant factor in the progression of this disease.^[18]^ In contrast, a mouse model of renal IRI has shown that an upsurge in mitochondrial AKT1 is essential for safeguarding renal structure and function. Interestingly, the inhibition of AKT1 expression can lead to an increase in oxidative stress injury, suggesting that AKT1 plays a critical role in mitigating the damaging effects of oxidative stress in this context.^[19]^ Thus, AKT1 may exert contradictory effects in renal disease. The JUN and FOS proteins are encoded by proto-oncogene c-Jun and proto-oncogene c-Fos, respectively. JUN proteins can form homodimers or heterodimers in combination with FOS proteins, which are involved in forming Activator Protein 1 (AP-1).^[20]^ AP-1 participates in the progression of multiple pathological processes, including fibrosis and inflammation.^[21]^ Activation of AP-1 may lead to glomerulosclerosis, interstitial fibrosis, and inflammation, which are essential causes of kidney injury.^[22]^ As one of the 3 MAPK signaling pathways, c-Jun amino-terminal kinase (JNK) can transmit extracellular signals to regulate cell proliferation, differentiation, and apoptosis. The JNK signaling pathway mediates the progression of CKD by participating in inflammation, fibrosis, oxidative stress, and nephrotoxicity.^[23]^ Studies have shown that G2/M blockade in renal tubular epithelial cells activates the JNK signaling pathway and promotes fibroblast factor secretion; blocking JNK signaling pathway activation inhibits TGF-β1-mediated renal fibrosis.^[24,25]^ The role of the TGF-β signaling pathway in the pathogenesis of CKD has been researched. According to Zhou, the knockdown of JUN and FOS genes in normal kidney RPTEC-TERT1 OAT3 cells or using AP-1 inhibitors significantly reduced TGF-β expression (*P* < .05). These findings indicate that AP-1 can mediate the activation of TGF-β signaling to promote the occurrence of renal fibrosis.^[26]^ As a member of the NF-κB protein superfamily, P65 (RELA) is mainly involved in inflammation, apoptosis, and tumor suppression, while its role in developing chronic kidney disease is gradually increasing. Tang identified P65 as a potential target for reducing inflammation and fibrosis in unilateral ureteral obstruction (UUO) mice, and inhibition of P65 expression could slow down the progression of AKI to CKD.^[27]^ In a gentamicin-induced kidney injury rat model, significantly increased NF-κB-P65 protein expression was observed by immunoblotting (*P *< .05), and HE staining showed dilated renal tubules and massive epithelial cell degeneration and necrosis.^[28]^ The tumor suppressor P53, a key transcription factor, is poorly expressed in normal cells, and activation of P53 stimulated by factors such as oxidative stress can lead to cell cycle arrest, apoptosis, DNA repair, and autophagy.^[29,30]^ Activation of P53 plays a crucial role in IRI models and cisplatin-induced acute kidney injury models, while P53 knockdown or inhibition can effectively protect kidney tissue from injury.^[31]^ As the main component of LWDH in treating CKD, quercetin exerts its anti-fibrosis and anti-inflammatory effects through multiple pathways. In the glucose-induced CKD cell model, quercetin can lead to reduced TNF-α levels and inhibit the activation of the TGFβ/smads pathway, suggesting its therapeutic potential in CKD.^[32]^ In an animal adenine-induced chronic renal failure model, quercetin reduced the expression of NLRP3, caspase1, p-PI3k, and p-Akt in kidney tissues, suggesting that quercetin can inhibit cellular apoptosis and achieve a protective effect.^[33]^ Hederagenin also exerted the same anti-fibrotic effect on fibroblast NRK-49 cells.^[34]^ Hyoxytopoly induction factor-1 (HIF-1) is a specific transcription factor that plays an important role in oxidation stimulation damage. The formation of AGES, reactive oxygen species (ROS), and inflammatory factors can activate HIF-1.^[35]^ In the IRI model and UUO model, the activation of HIF-1 prevents renal fibrosis, while its inhibition increases the degree of renal fibrosis.^[36]^

Secondly, most of these essential targets are related to inflammation. Inflammation is a major mechanism for the immune system to adapt and defend against external stimuli or harm. In CKD, inflammation is a critical factor that can impair kidney repair and lead to the development of renal fibrosis. Studies have shown that high levels of serum TNFα, IL-6, and other inflammatory factors in CKD patients are closely linked to the loss of renal function over time.^[37]^ Multiple causes of inflammation have been reported in CKD, including reduced GFR, increased production or reduced clearance of pro-inflammatory factors, oxidative stress, uremia, infection, dialysis, etc.^[38]^ IL-6 is a soluble protein produced by T cells that activates B cells to differentiate into antibody-producing cells and is closely associated with inflammation.^[39]^ IL-6 is a risk factor for many complications of CKD, such as anemia, pruritus, and coronary syndrome.^[40–42]^ In a recent study, IL-6 was found to mediate increased expression of FGF23, leading to increased morbidity and mortality in AKI and CKD.^[43]^ TNF-α is mainly secreted by macrophages, NK cells, T cells, and B cells, and mainly mediates the development of inflammation and cell death.^[44]^ TNF-α inhibitors reduced cisplatin-induced renal structural and functional damage. At the same time, TNF-α-deficient mice were resistant to cisplatin nephrotoxicity. TNF-α was also involved in ciprofloxacin-induced nephrotoxicity, suggesting the essential role of TNF-α in the pathogenesis of renal injury.^[45,46]^ As an anti-inflammatory factor, IL-10 can protect the kidney by suppressing excessive inflammatory responses, modulating immunosuppression, delaying fibrosis, and promoting repair.^[47]^ IL-10 knockout mice showed lipid accumulation, elevated cholesterol, glomerulosclerosis, tubular and interstitial fibrosis, immune cell infiltration, and elevated serum inflammatory factor expression levels in the kidney and serum after 12 weeks of high-fat diet feeding, suggesting that IL-10 is involved in obesity-associated renal disease.^[48]^ IL-10 also delays the progression of UUO-induced renal fibrosis by inhibiting endoplasmic reticulum stress.^[49]^ IL-1a and IL-1b belong to the IL-1 superfamily. IL-1a can readily release pro-inflammatory alarmins during cell necrosis, causing local tissue inflammation, and IL-1b promotes systemic inflammation primarily by initiating acute phase response proteins (such as C-reactive protein) in the liver, activating endothelial cells, triggering fever, mobilizing neutrophils (leukocytosis), and activating all types of leukocytes.^[50]^ A clinical study shows that IL-1b inhibitors reduce the incidence of major adverse cardiovascular events in patients with high-risk atherosclerosis in CKD.^[51]^

Thirdly, in our study, GO enrichment analysis showed that LWDH intervened in CKD mainly through their involvement in oxidative stress. The imbalance of the body’s oxidative and antioxidant systems induces oxidative stress. The oxidative system mainly consists of ROS, which includes superoxide, hydroxyl radicals, and hydrogen peroxide. Superoxide can be catalyzed by superoxide dismutase into hydrogen peroxide, and hydrogen peroxide is dismutated into hydroxyl radicals through redox reactions, which is the most active ROS.^[52]^ The antioxidant system includes antioxidant enzymes, glutathione peroxidase, and catalase, which are responsible for scavenging ROS.^[53]^ Furthermore, oxidative stress injury is associated with the development of many diseases, including CKD. Studies have shown that AKI and CKD lead to increased levels of oxidative stress and that advanced oxidative protein products and fructosamine can be used as biomarkers for CKD.^[54]^ Reducing ROS levels can mediate the inhibition of the TGF-β signaling pathway and thereby reduce the extent of renal fibrosis in UUO mice.^[55]^ Clinical studies have found that as CKD progresses, patients’ serum malondialdehyde and oxidized glutathione levels increase, whereas glutathione levels decrease, suggesting increasing levels of oxidative stress and significantly lower antioxidant levels in the body.^[56]^ Advanced-glycation end products (AGEs) are heterogeneous molecules obtained by post-translational non-enzymatic modification of macromolecules, including proteins and nucleic acids by other types of sugars such as glucose or fructose. In various age-related diseases, binding AGEs to RAGEs can promote oxidative stress and thus enhance inflammatory responses.^[57]^ An RCT revealed that patients’ AGEs/sRAGE levels gradually increased as CKD progressed, suggesting that AGEs/sRAGE may be a risk factor for CKD.^[58]^ In addition, AGEs levels were negatively correlated with the physical condition and vitality of dialysis patients, and the accumulation of AGEs in the gastrocnemius muscle in the 5/6 nephrectomy mouse model was closely associated with morphological abnormalities and altered mitochondrial function.^[59]^ In RAGE knockout mice, the time to CKD-induced arterial thrombosis was significantly reduced, suggesting that AGEs-RGEs are involved in this process and that blocking RAGE may be a mechanism to protect against CKD-induced cardiovascular disease.^[58]^

In addition to the inflammation, fibrosis, and oxidation stress discussed above, many factors can participate in the occurrence and treatment of CKD, such as apoptosis. Apoptosis is an important cell death mechanism in the body. Eukaryotic cell are mainly based on external apoptosis pathways, internal mitochondrial pathways, Granzyme B -mediated cell apoptosis pathways, and internal quality network stress channels. Granzyme B, Cysteine proteases (Caspases) and BCL-2 protein molecules are involved in the apoptosis mechanism. Cell pigmentation C is released from mitochondria to the cytoplasm through the BAX/BAK hole to activate cysteine proteases that start the apoptotic process, such as Caspase-9, and then activate Caspase-3 to exert the apoptosis effect.^[60]^ Apoptosis is observed in AKI and CKD, which may be the main mechanism responsible for acute and chronic tubular cell and foot cell loss.^[61]^ Increased caspase-3 activity, Bax expression, and Bax/Bcl ratio were observed in renal dysfunctional rats.^[62]^ Cadmium-induced apoptosis in chicken kidney cells by promoting the increase of Bax, caspase-9, and caspase-3 levels at a dose of 2.15 g/kg.^[63]^ These studies all indicate that LWDH may treat CKD by inhibiting apoptosis, which was confirmed by our protein interaction analysis results.

The current uncertainty surrounding therapeutic targets for CKD highlights the urgent need for new strategies. Our study has identified several potential targets for the treatment of CKD, providing a fresh perspective for the development of new drugs. Despite the impressive effectiveness of LWDH, limited research has been conducted on its mechanism of action. Using network pharmacology and molecular docking studies, this report attempts to elucidate the mechanism of action of LWDH on CKD. Nevertheless, the limitations of this study should be acknowledged. Firstly, due to the real-time updates of the database, we were unable to obtain the latest information on biological activity ingredients and targets, which may be crucial in studying LWDH for CKD. Secondly, while the molecular docking results validated the predictions, further animal or cell experiments are necessary to explore the predicted active ingredients. Despite considerable research in this area, experimental verification is essential to supplement the evidence of LWDH for CKD and is also necessary to further determine the dose-effect relationship of LWDH in the treatment of CKD. Lastly, while our results predict target proteins and signal channels, there may be other pathways through which LWDH can treat CKD. Our future research will explore these pathways and their relationships. Our ultimate goal is to contribute to the development of more effective and targeted therapies for this debilitating disease.

## 5. Conclusion

The multiple active components present in LWDH make it an effective treatment for CKD. LWDH effectively addresses the complex pathophysiology of CKD by acting on multiple targets, participating in multiple biological processes, and activating various signaling pathways. The central components of LWDH, including quercetin, stigmasterol, kaempferol, beta-sitosterol, tetrahydroalstonine, kadsurenone, hederagenin, hancinone C, diosgenin, and sitosterol, play a vital role in regulating the response to chemical stimuli, reactive oxygen species metabolism, and key signaling pathways such as AGE-RAGE, IL-17, and TNF pathways for CKD. These components interact with central targets such as AKT1, JUN, and IL6 to produce therapeutic effects. Through network pharmacology analysis, this study identified the potential mechanisms of LWDH in treating CKD. The results indicate the multi-component-multi-target-multi-pathway pharmacological effects of LWDH on CKD, highlighting its significant potential for the treatment of chronic kidney disease and warranting further investigation.

## Author contributions

**Conceptualization:** Xi Xie, Hongjun Lou, Xi Gao.

**Data curation:** Xi Xie, Hongjun Lou, Ye Shi, Guang Gan, Hanqing Deng.

**Formal analysis:** Hanqing Deng.

**Methodology:** Xi Xie, Ye Shi, Guang Gan, Hanqing Deng, Mingfang Meng.

**Supervision:** Xi Gao.

**Visualization:** Xi Xie, Hongjun Lou, Xinwei Ma.

**Writing – original draft:** Xi Xie, Hongjun Lou, Ye Shi.

**Writing – review & editing:** Hongjun Lou, Xinwei Ma, Mingfang Meng.
